# 

**Published:** 1892-03-12

**Authors:** 


PRICE ONE PENNY.

No. 285, Vol. XI.—March 12th, 1892.

Registered at the General Post Office as a Newspaper for transmission in the United Kingdom and Abroad.]

WITH EXTRA NURSING SUPPLEMENT.
Per Annum, 6s. 6d., post free.
Monthly Parts, 6d.; post free, 8d.
Ditto per Annum, 8s., post free

### THE HOSPITAL

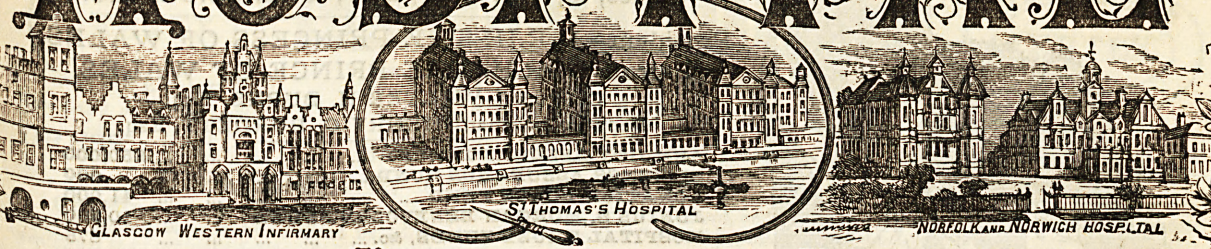

A WEEKLY JOURNAL OF

### Science, Medicine, Nursing, and Philanthropy.

SATURDAY, MARCH 12th, 1892.

**An American Sensation** ... 285
**Passing Topics.**—Exeunt ... 286
**The Doctor's Armchair.**—A Persecuted Hospital ... 287
**Some Scientific Aspects of Insanity.** XVI.—The Insanities of the Various Periods of Life ... 288
**The History and Capabilities of Herbal Simples.** By W. T. Fernie, M.D. XXXVIII.—Feverfew ... 288
**Everybody's Page** ... 289
**Notes and News** ... 290
**Annotations.**—The Care of the Feeble-minded—A Little Bacteriology—A Boy Murderer ... 292
**General Charities—Scraps and Gleanings** ... 291
**The Practitioner's Mirror and Retrospect—**
Medicine.—The Chloroform and Ether Controversy, 293; Treatment of Diphtheria by Sub-membranous Injections 294
Practitioners' Bookshelf.—Diseases of the Ovaries and Fallopian Tubes ... 294
New Drugs, Appliances, and Things Medical.—Newly Invented Hip Disease Chair ... 295
**Talks About Food.** III.—How the Rich Live ... 296
**The Student of Medicine.**—Appointments—Vacancies—Notes from the Schools—Pass Lists—Athletics.

EXTRA SUPPLEMENT.—THE NURSING MIRROR.

**En Passant.**—A Nurse in Bermuda—An Influenza Story—Poor-Law Nurses—St. Mary's Nurses' Home—Short Items—The Worcester Institution ... cxxxix

**On the Nursing of Children.** V.—Splints ... cxl

**Presentations** ... cxl

**Appointments** ... cxl

**Nursing Uniforms.** II.—Guy's Hospital ... cxli

**Death in Our Ranks** ... cxli

**Wants and Workers** ... cxli

**The Royal National Pension Fund for Nurses** ... cxli

**Off Duty.**—My Kitten ... cxliv

**Notes and Queries** ... cxliv

